# Primary Pleural Tuberculosis Presenting With Noncaseating Granulomas: A Diagnostic Challenge

**DOI:** 10.1155/crdi/6448395

**Published:** 2026-05-06

**Authors:** Maria Akiki, Chebly Dagher, Jessica Abrantes-Figueiredo

**Affiliations:** ^1^ Department of Internal Medicine, University of Connecticut, Farmington, 06030, Connecticut, USA, uconn.edu; ^2^ Division of Infectious Diseases, Saint Francis Hospital and Medical Center, Hartford, 06105, Connecticut, USA, stfranciscare.org

**Keywords:** *Mycobacterium tuberculosis*, noncaseating granuloma, pleural biopsy, pleural tuberculosis, thoracoscopy

## Abstract

Tuberculous pleural effusion is the second most common form of extrapulmonary tuberculosis after tuberculous lymphadenitis. Despite its frequency, diagnosis remains challenging due to its paucibacillary nature, and confirmation often requires invasive procedures. We report a 26‐year‐old male with no significant medical history who presented with persistent dyspnea, cough, and left‐sided chest pain after recent travel to Pakistan. He denied known tuberculosis exposure. Imaging demonstrated a left pleural effusion, and thoracentesis revealed a lymphocytic exudate with elevated adenosine deaminase. Pleural fluid acid‐fast bacilli smear and tuberculosis polymerase chain reaction were negative. Given the high clinical suspicion for tuberculosis, the patient underwent thoracoscopy with pleural biopsy, which demonstrated noncaseating granulomas. Although the histopathological findings were atypical, empiric antituberculous therapy was initiated. One month later, pleural biopsy cultures grew *Mycobacterium tuberculosis*, confirming the diagnosis. Pleural tuberculosis remains difficult to diagnose, as conventional microbiological tests are highly specific but have limited sensitivity. This case highlights the critical role of pleural biopsy in guiding early management and underscores that empiric therapy should be strongly considered when clinical suspicion and epidemiologic risk factors are high, even if initial diagnostic studies are negative.

## 1. Introduction

Tuberculous pleural effusions are a well‐recognized manifestation of extrapulmonary tuberculosis (TB), with prevalence rates ranging from 3% to 30%, with the highest rates observed in developing countries [1]. Among extrapulmonary TB cases, pleural tuberculosis (pTB) is the second most frequent form after tuberculous lymphadenitis, contributing to 15%–25% of TB cases globally [2]. Its epidemiological patterns closely resemble those of TB in general, with a greater incidence in males, individuals with weakened immune systems, and socioeconomically disadvantaged individuals [3]. The clinical spectrum of pTB varies widely, ranging from simple effusions that resolve without intervention to purulent TB empyema that can lead to chronic complications, as well as lipid‐associated pleural effusions [4]. In cases where no evident lung disease is present, *Mycobacterium tuberculosis* (Mtb) is thought to enter the pleural space following the rupture of a caseous focus situated just beneath the pleura, allowing bacterial dissemination into the pleural cavity [5]. Both experimental models and clinical observations indicate that this process triggers an initial influx of neutrophils and monocytes, followed by a prolonged accumulation of presensitized T‐lymphocytes, ultimately resulting in persistent pleural effusion [6]. The immune response in most TB‐related pleural effusions is dominated by memory T‐helper 1 (Th1) cells, which are believed to have been activated during the initial infection and subsequently migrate to the affected pleural area [6]. However, around 10% of pleural effusions in human studies exhibit a neutrophil‐predominant profile [7]. TB‐related pleural effusions are typically unilateral [8]. Diagnosing pTB necessitates detecting Mtb in pleural fluid, tissue or identifying caseating granulomas on a pleural biopsy, preferably in conjunction with acid‐fast bacilli (AFB) [4]. The recruitment of IFN‐γ‐producing T‐cells typically triggers a robust immune response against TB, contributing to the low bacterial burden in most tuberculous effusions, which in turn makes achieving a definitive diagnosis challenging [2]. Also, it is important to recognize that TB may also present with noncaseating granulomas, complicating the diagnostic process by mimicking other granulomatous diseases [9]. A review of 103 pTB cases over eight years reported microbiological confirmation in only 15.5% of patients [10]. Factors that increase the likelihood of an accurate diagnosis include residence in regions with a high TB burden, previous exposure to TB, and impaired immunity [11]. Thoracoscopy remains the most effective diagnostic approach, as it allows for the collection of substantial pleural tissue samples for histological and microbiological testing [12]. The management of pTB mirrors that of pulmonary TB; however, drug delivery to the pleural space may be less effective in cases with complicated effusions. In some instances, drainage may be required as a therapeutic intervention [13].

## 2. Case Presentation

A 26‐year‐old male presented with 2 weeks of persistent shortness of breath, cough, and left‐sided chest pain. He denied fever, chills, night sweats, or weight loss. He had no known chronic medical conditions, was not taking any regular medications, and had no history of immunosuppressive therapy. He was born in Pakistan, lived in Connecticut for two years, and visited Pakistan the previous summer. He denied TB contacts and had a previously negative purified protein derivative test. He works as a mechanic and has a history of smoking. He had initially sought care a week prior, at which time a chest X‐ray showed a left basilar opacity with pleural effusion, and he was discharged on azithromycin and amoxicillin. However, his symptoms worsened, prompting his return. On admission, he was hemodynamically stable, afebrile, and maintained adequate oxygen saturation on room air. Physical examination revealed a well‐appearing patient in no acute distress. His cardiac examination was unremarkable, while pulmonary auscultation demonstrated rales in the left lower lobe. The abdominal exam was soft and nontender, and there was no evidence of lower extremity edema. Laboratory tests showed a white blood cell count of 9.8 × 10^3^/μL, hemoglobin of 13.1 g/dL, platelet count of 510 × 10^3^/μL, aspartate aminotransferase of 52 U/L, alanine aminotransferase of 64 U/L, and creatinine of 0.9 mg/dL. A computed tomography scan of the chest revealed a large, complex left pleural effusion with rim‐enhancing pleural thickening (Figures 1(a) and 1(b)). He was started on vancomycin, cefepime, and metronidazole for suspected complicated pneumonia. A thoracentesis was performed, showing a lymphocytic‐predominant effusion with 4080 nucleated cells/μL (5% polymorphonuclear cells), 3352 red blood cells/μL, an elevated adenosine deaminase (ADA) of 48 U/L, lactate dehydrogenase of 611 U/L, protein of 6.1 g/dL, and glucose of 64 mg/dL, raising suspicion for tuberculous pleuritis. Cytology was negative for malignancy, and pleural fluid cultures grew *Enterococcus faecalis*. AFB smear and TB polymerase chain reaction on pleural fluid and sputum were both negative. Given his history of travel to Pakistan and a positive interferon‐gamma release assay, he underwent left thoracoscopy with pleural biopsy, which showed noncaseating granulomatous inflammation with a background of lymphoplasmacytic infiltration (Figures 2(a) and 2(b)), though periodic acid‐Schiff and AFB stains were negative. Given these findings, broad‐spectrum antibiotics were discontinued, and he was started on amoxicillin‐clavulanate for *E. faecalis*, along with rifampin, isoniazid, pyrazinamide, and ethambutol for presumed pTB. His hospitalization was complicated by bilateral deep vein thrombosis and pulmonary embolism, for which he was started on therapeutic enoxaparin. A month later, cultures from the pleural biopsy confirmed Mtb, solidifying the diagnosis of primary pTB. With continued rifampin, isoniazid, pyrazinamide, and ethambutol therapy, the patient showed significant clinical improvement, with the resolution of respiratory symptoms and improved functional status. The patient reported symptomatic improvement and satisfaction with treatment.

FIGURE 1(a) Chest CT (lung window) showing a large, complex left pleural effusion. (b) Chest CT (mediastinal window) demonstrating rim‐enhancing pleural thickening associated with the effusion.

**Figure:**
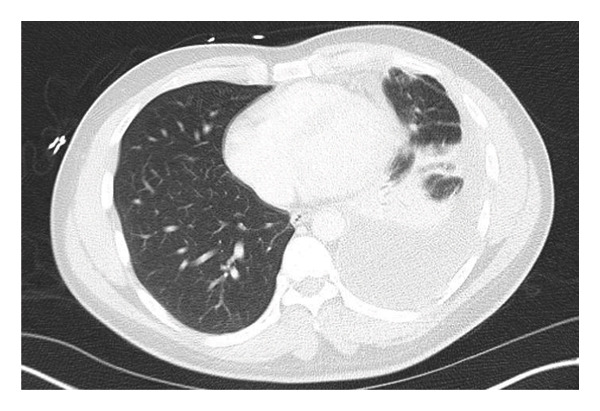
(a)

**Figure:**
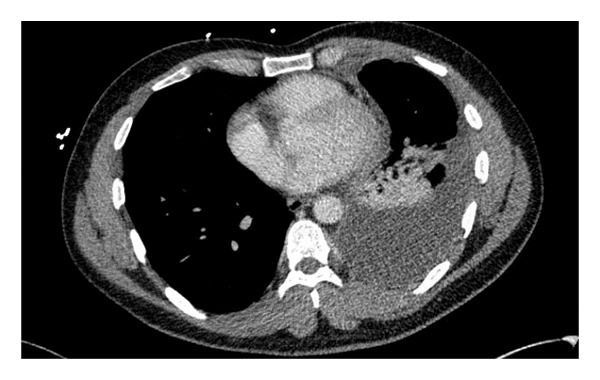
(b)

FIGURE 2(a) Thoracoscopic pleural biopsy showing noncaseating granulomatous inflammation with lymphoplasmacytic infiltration on hematoxylin and eosin stain. (b) Thoracoscopic pleural biopsy showing noncaseating granulomatous inflammation with lymphoplasmacytic infiltration on hematoxylin and eosin stain at a different field.

**Figure:**
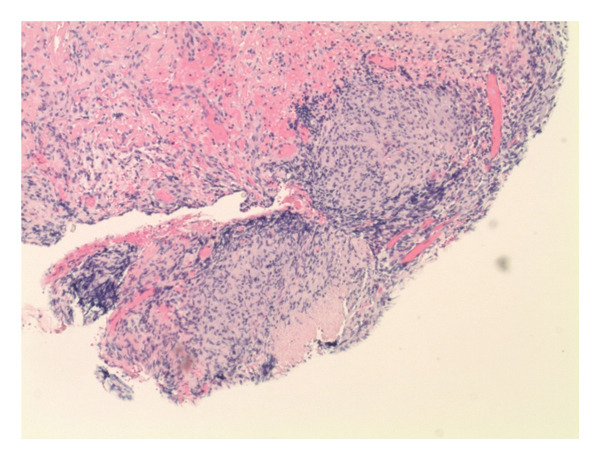
(a)

**Figure:**
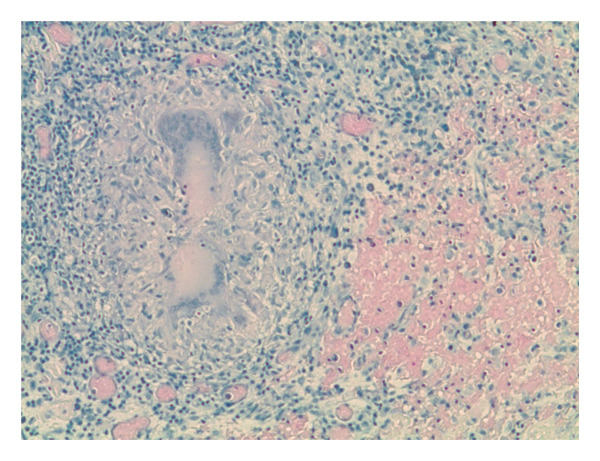
(b)

## 3. Discussion

TB remains a significant global health concern, with approximately 8.2 million patients diagnosed in 2023 alone, according to the World Health Organization [14]. Pulmonary TB is classically associated with cavitary lesions in the upper lobes, yet pleural involvement without pulmonary parenchymal disease is a rare presentation [13]. Given the paucibacillary nature of the disease, diagnosis remains a challenge [13].

Certain studies indicate that an ADA level exceeding 45 to 60 units/L has a sensitivity of 100% and a specificity of up to 97% for diagnosing tuberculous pleural effusion [8]. Nucleic acid amplification (NAA) techniques for diagnosing tuberculous pleural effusion in HIV‐negative patients demonstrate high specificity but comparatively low sensitivity [15]. A meta‐analysis of 18 studies reported that the GeneXpert Mtb/rifampin assay, when compared to culture, had a sensitivity of 46% and a specificity of 99% in pleural fluid [16]. Given these diagnostic constraints, pleural biopsy remains a valuable tool, with studies showing a diagnostic yield ranging from 60% to 95% [17, 18]. Additionally, pleural biopsy cultures yield positive results in approximately 40% to 80% of cases, while the histopathological examination identifies granulomas in 50% to 97% of cases [19]. Inchingolo et al. proposed a structured diagnostic algorithm for pTB that integrates epidemiologic risk factors, pleural fluid biomarkers, molecular testing, and early pleural biopsy to optimize diagnostic accuracy [20]. Their framework supports the early use of thoracoscopic biopsy in high‐suspicion cases with negative pleural fluid studies, consistent with the approach taken in our patient [20]. Histopathological findings in TB typically reveal caseating granulomas, which are considered highly characteristic of the disease, reinforcing its role in confirming tuberculous pleuritis [9]. However, noncaseating granulomas, as seen in our patient, are rare in TB and more commonly associated with conditions such as sarcoidosis, fungal infections, and granulomatous inflammatory diseases [9, 21]. This atypical histology may delay diagnosis and lead to consideration of alternative etiologies. Despite negative AFB smears and an atypical granulomatous pattern, a high clinical suspicion of TB, based on risk factors such as travel history, positive interferon‐gamma release assay, and elevated ADA in pleural fluid, warranted early initiation of antituberculous therapy.

## 4. Conclusion

pTB may present with atypical findings such as noncaseating granulomas and negative initial microbiologic tests, which can make the diagnosis challenging. In these situations, other causes of granulomatous disease, including sarcoidosis and fungal infections, should be carefully considered before starting empiric treatment. When the clinical picture and epidemiologic risk factors raise strong suspicion for TB, pleural biopsy plays an important role in establishing the diagnosis, and empiric antituberculous therapy may be started while awaiting culture results. Final microbiologic confirmation remains important, and patients should be followed closely to ensure clinical improvement and completion of therapy.

## Author Contributions

Maria Akiki was primarily responsible for the conception of the manuscript, performed the literature review, and wrote the initial draft. Chebly Dagher contributed to the literature review and assisted in manuscript writing. Jessica Abrantes‐Figueiredo supervised the clinical management of the patient, provided critical revision of the manuscript, and offered overall oversight.

## Funding

This research did not receive any specific grant from funding agencies in the public, commercial, or not‐for‐profit sectors.

## Disclosure

The authors disclose that a version of this manuscript has been made publicly available as a preprint on the Social Science Research Network platform prior to submission for peer review. The preprint has been assigned a digital object identifier (DOI: 10.2139/ssrn.5309009)^21^. The preprint version has not undergone peer review. The present manuscript represents the version submitted for formal peer review and consideration for publication and has not been previously published in a peer‐reviewed journal. All authors reviewed and approved the final manuscript.

## Consent

Written informed consent was obtained from the patient for the publication of this case report. A copy of the written consent is available for review by the journal’s editorial office upon request.

## Conflicts of Interest

The authors declare no conflicts of interest.

## Data Availability

The data supporting the findings of this study are included within the article. Additional details are not publicly available to protect patient confidentiality but may be made available from the corresponding author upon reasonable request.
